# Teen Alcohol Use and Social Networks: The Contributions of Friend Influence and Friendship Selection

**DOI:** 10.4172/2329-6488.1000224

**Published:** 2015-10-19

**Authors:** Jacob E Cheadle, Katrina M Walsemann, Bridget J Goosby

**Affiliations:** 1 The University of Nebraska-Lincoln, 737 Old father Hall, Lincoln, NE 68588-0324, USA; 2 Department of Health Promotion, Education, and Behavior, Arnold School of Public Health, 800 Sumter Street, Room 216, Columbia, SC 29208, USA; 3 The University of Nebraska-Lincoln, USA

## Abstract

**Background:**

We evaluated the contributions of teen alcohol use to the formation and continuation of new and existing friendships while in turn estimating the influence of friend drinking on individuals’ regular use and heavy drinking.

**Method:**

Longitudinal network analysis was used to assess the mutual influences between teen drinking and social networks among adolescents in two large Add Health schools where full network data was collected three times. Friendship processes were disaggregated into the formation of new friendships and the continuation of existing friendships in a joint model isolating friendship selection and friend influences.

**Results:**

Friends have a modest influence on one another when selection is controlled. Selection is more complicated than prior studies suggest, and is only related to new friendships and not their duration in the largest school. Alcohol use predicts decreasing popularity in some cases, and popularity does not predict alcohol consumption.

**Conclusion:**

Intervention efforts should continue pursuing strategies that mitigate negative peer influences. The development of socializing opportunities that facilitate relationship opportunities to select on healthy behaviors also appears promising. Future work preventing teen substance use should incorporate longitudinal network assessments to determine whether programs promote protective peer relationships in addition to how treatment effects diffuse through social networks.

## Introduction

Friends and peers are key to whether, when, and how much adolescents drink alcohol [1–3] and are therefore central to prevention [4–7]. By 12^th^ grade nearly 50% of teens report being frequently with others drinking to get high, 75% indicate that one or more friends drink until drunk routinely [8], and over 80% drink to have a good time with friends [9]. Because drinking impairs cognitive functioning and judgment [10], promotes risky behaviors [11–13], and leads to accidents and mortality [14], understanding how friendships shape— and are shaped by—drinking is a critical public health issue [8].

In service to programmatic efforts to reduce teen drinking, researchers have sought to determine the magnitude of *friend influence* [15] by linking friends’ drinking to individual drinking [16]. One central challenge has been the inherent difficulty in accounting for *friend selection*, the process by which peers become friends, when estimating friend influence [17,18]. Without accounting for selection, it is impossible to accurately determine whether one’s drinking is influenced by how much friends drink, or whether one’s drinking reflects homophily [19]—the extent to which “birds of a feather flock together” [20]. Individuals may seek out others who drink like they do, or select into environments where drinkers socialize together [21], rather than adjusting behaviors to be more like those of friends’ [22,23].

Longitudinal social network analysis using methods [24] to decompose teen alcohol use into separate selection and influence components finds roles for both processes [25]. Although there is disagreement about when selection and influence each emerge in importance over adolescence, both factors contribute to the correlation between friendship networks and alcohol use [26–28]. We extend this novel line of research by jointly estimating the contributions of friendships to adolescents’ drinking, and how alcohol use contributes to whether new friendships form and existing friendships continue [21,29].

We use network analysis because self-reports are unreliable and inflate influence estimates [30,31]. Social network measures capture connections between each adolescent and other students based upon reported friendships, directly capturing the friendship patterns of all youth in the same school [32,33]. Because all adolescents in a school are assessed, each reports on his or her own behavior, so friend estimates are not subject to the “same-source bias” problem that confounds influence estimates in traditional observational studies [34].

We assess how alcohol use contributes to drinking homophily with both individual drinking behavior and friendship selection modeled as mutually influencing processes to account for the inherent endogeneity of influence and selection [18,23]. We assess the roles of teen popularity (receiving friendship nominations), sociability (nominating friends), and friend influence (average friend alcohol use) on drinking alcohol, while controlling for selection [35]. Simultaneously, we assess the role of drinking in connecting adolescents to one another via new and existing friendships, and thus in shaping the friendship network as individual and friend drinking changes over time.

## Methods

### Participants

We use National Longitudinal Study of Adolescent Health’s (Add Health) wave 1 in-school survey (observation point 1), and the wave 1 and 2 in-home surveys (observation points 2 and 3) for up to three observation points. Add Health is a cluster stratified longitudinal study of 7–12^th^ grade students in 1994. Add Health researchers obtained parent and child consent and provide de-identified data to other researchers under approved security protocols [36]. All procedures for this study, including data security protocols for working with the restricted de-identified data, were approved by the University of Nebraska-Lincoln IRB.

We use a subset of the 16 schools where friendship data was collected at each observation point. Of these 16 schools, two are mid-sized or larger (*n*>1000), and 14 are small (*n*<300). We analyze network data from the two largest schools, because the other smaller schools were either special education or middle schools, or because a network sampling error at observation 2 restricted participants to nominating only one female and one male friend rather than up to 5 of each (about 5% in the schools we use and over 50% in other schools; we include an indicator for this subset of students). The resulting sample was 2,296 adolescents; 1,531 in the large, racially heterogeneous high school, which is commonly referred to as “Jefferson High School”, and 765 in the middle-sized predominantly white high school, commonly referred to as “Sunshine High School” [37]. Sunshine was 7% nonwhite, and Jefferson was 6% white, 23% black, 39% Hispanic, and 32% Asian. The network response rates are acceptable for social network analysis [38]. Approximately 65–97% of teens provided information on at least one friend within the network at each wave, and 86% provided at least one nomination at 2+ waves. Missing data were handled within the estimation procedure with the composition change method developed for longitudinal network models [39], so that all youth were included in the analysis and allowed to enter the study later or leave. The sample was limited to youth with at least two drinking observations, and missing drinking/attribute data is model imputed using standard procedures [38,40].

### The close friendship network

The *close friendship network matrix* captures the system and structure of relationships among adolescents at each observation point and so plays two roles in our models: it is both a primary endogenous variable for modeling selection, and it captures the relationships necessary for estimating friend influence [41]. Networks are constructed from up to five male and five female friend nominations from the school roster at each wave separately. The nomination question, with male nominations as the example, was worded as “List your closest male friends. List your best male friend first, then your next best friend, and so on. Girls may include boys who are friends and boyfriends.”

### Alcohol use

*Alcohol use frequency* predicts and is predicted by the friendship network. It is based on the question, “During the last 12 months, on how many days did you drink alcohol?” This item is a standard intensity assessment measured on a seven-point scale with values for never drinks, once or twice in the last year, once a month or less, 2–3 days a month, 1–2 days a week, 3 to 5 days a week, and every day or almost every day [22]. Due to sparse distributions in the upper categories, we top-coded alcohol use at the sixth category. Drinking similarity, which ranges between 0 (dissimilar) to 1 (perfectly similar), in the network is modest between friends: 0.55 (Sunshine) and 0.61 (Jefferson). In order to understand how close friendship is linked to heavier drinking, we also model *drunkenness frequency* (friend similarity: 0.65 [Sunshine] and 0.75 [Jefferson]) with the same categories as for alcohol use, from the following question: “During the past twelve months, how often did you get drunk?”

### Control variables

*Female* is included to reflect sex-stratification in adolescent friendships [42], *grade level* and *race/ethnic background* [43,44], which is captured in the model with an indicator for whether or not dyads are of the same race/ethnic background in the selection model, and by black, Hispanic, and Asian indicators in the behavioral model (Jefferson) or an indicator for non-white (Sunshine) in the selection model, are all included. Adolescents self-stratify socioeconomically [45], so *parent education* (observation 2) is included as: did not graduate from high school, graduated from high school, some higher education, graduated from college, and obtained advanced schooling.

Three additional factors related to alcohol use are included. The first, drawn from observation 2, is *parent drinks alcohol* (1=never to 6=nearly every day). Parents model alcohol use [46] and friend-parent similarity is higher than chance [47]. Because access may support alcohol use selectivity, *whether alcohol is easy to get* (observation 2) is measured from the question “Is alcohol easily available to you in your home?” Finally, whether the youth is a *regular smoker* (ever smoked at least one cigarette a day for at least 30 days) is a time-varying covariate that influences friend selection [48] and is correlated with alcohol use [9,11]. The final control is a time-varying (observations 1 and 2) off-list nominations count capturing close friendships outside of school.

### Statistical analysis strategy

The analysis uses Snijders and colleagues’ [24,35,41] stochastic actor-based (SAB) network model. Parameters reflect changes in network statistics and drinking across waves using a method of moment’s estimator summarizing network-behavior configuration changes between observations. Agent-based simulations update parameters, estimate uncertainties, and provide an interpretational framework. The data-constrained simulation model decomposes network changes into sequential transitions in either one tie or drinking for a randomly selected adolescent. Change opportunities are governed by rate parameters determining the simulation steps needed to reproduce changes in the observed data between observations.

Friendship selection captures friendships over time. This model dimension specifies network structure and attributes on change/stability in friendship status [49]. Selection is operationalized with four parameters to discriminate between the different ways that drinking affects friendships. The *alter* effect captures the extent to which teens are chosen as friends based on their alcohol use (popularity) and the *ego* effect reflects whether drinking is related to nominating more friends (sociability). The *ego-alter interaction* term, the primary selection effect, is a dyadic effect expressing an increasing logit of friendships among higher drinkers. This effect is included first as a baseline term capturing the presence of a friendship or not. It is then disaggregated to reflect (b) the formation of new friendships, and (c) the continuation of existing friendships [29]. Other included network controls/statistics appear in (Table 1).

The friend influence model is similar to ordinal logistic regression [50]. In addition to background controls, we include the following parameters (see Table 1): *In degree* expresses how many friendship nominations an adolescent received and measures popularity [51]. *Out degree* records nominations of friends, reflecting sociability. *Average alter* is the average alcohol use of the adolescent’s friends and is the primary social influence measure [35]. Main effects for control variables and parameters for the alcohol use distribution are also included.

## Results

### Descriptive statistics

Descriptive statistics are presented in Tables 2 and 3. Alcohol use and drunkenness means are stable over time, and are slightly higher in Sunshine than Jefferson, even though similar proportions of youth report that alcohol is easy to get. Supplementary analyses indicate that approximately 40–50% of adolescents increased/decreased their regular alcohol use in both schools, but only 30% either increased/decreased the frequency with which they got drunk. The average number of friends nominated in Sunshine decreased from nearly 6 at observation 1 to 3.5 at observation 3, and from 3.6 to fewer than 2 in Jefferson Table 3. Jaccard distances indicate that the amount of network change is sufficient for longitudinal network modeling [52] (Tables 2 and 3).

### Regular alcohol use

Focal alcohol use influence and selection parameters are presented in Table 4 for average effects across schools, by school, and with t-ratios comparing Sunshine and Jefferson (full results available online). Average results were estimated by combining both schools into a single analysis with both schools joined into a multigroup sequential analysis [53]. Coefficients are *logits*.

The first panel contains results from two models with selection and influence estimated independently. The three inferences are first that drinking is differentially related to popularity by school; it is related to increased popularity in Sunshine (b=.35), but lower popularity in Jefferson (b=−0.057; t=3.57). In Sunshine, for example, each level of alcohol use increases the odds of receiving a friendship nomination by 4% (exp[.035]=1.04). Second, drinking frequency predicts friendship selection. Two adolescents with drinking levels one unit above the mean have friendship odds 11% larger (exp [0.104]=1.11) than for two teens with average drinking. Third, average alter in the influence model indicates that higher friend use is associated with increasing individual use. For example, in Sunshine, the odds that a teen with average use but whose friends are on average 1-unit above the mean has odds of increasing use that are 30% larger (exp (0.266)=1.3) than if those friends also had average use (Table 4).

Model 3 disaggregates the ego-alter selection term into differences in the formation of new friendships and continuation of already existing relationships. Drinking predicts forming new friendships and friendship continuation in Sunshine, but only friendship continuation in Jefferson. This pattern persists in Model 4 where influence is controlled. Notably the influence term is similar to the Model 2 results (panel 1), indicating that influence is not strongly biased by selection. Consistency in selection similarly suggests that influence and selection both matter substantively but are largely statistically independent.

Model 5 add the measures of in degrees (popularity) and out degrees (sociability) to the influence model, along with measures of network closure to the selection model (see Table 1). Control variables appear in Model 6. Selection and influence results are consistent across models. Drinking is related to popularity (alter) in Sunshine but not Jefferson, new friendships in Sunshine but not Jefferson (ego-alter, new), continuation of existing friendships in both schools (ego-alter, old), and that influence is an important process in both. Drinking selection is never related to increased friend nominations (sociability), and neither popularity (in degree) nor sociability (out degree) predicts drinking changes, indicating that popularity does not predict drinking changes, or that being socially active predicts use.

### Drunkenness model results

A parallel model series is shown in Table 5 for drunkenness frequency. The results are similar to alcohol use frequency, but also have important differences. First, drunkenness is never related to popularity in Sunshine, suggesting some nonlinearity in the returns to drinking in that setting, and even greater associated negativity in Jefferson. Second, drinking selection in both schools reflects the tendency for heavier drinkers to form new friendships, but is not related to old friendship continuation. Third, individual drunkenness changes are subject to friend influences, just as with drinking frequency. Notably, there are fewer significant effects in Jefferson, mostly as a result of decreasing precision with increasingly complicated models. E.g., the size of the ego-alter interaction (new) is the same across schools, but is not significant in Jefferson. The influence effect is also of similar size in the joint analysis and is statistically significant due to greater precision (Table 5).

## Discussion

Adolescent drinking, like other behaviors, predicts friendships, but is also influenced by those relationships [54,55]. We accounted for selection when estimating friend influences on drinking, but also extended prior selection research [26–28] by assessing how drinking leads to new friendships and the continuation of existing friendships. Prior studies have generally not distinguished between friendship formation and continuation [21,29], limiting our understanding about how drinking contributes to friendship selection and thus how adolescent social networks are configured.

The inability of prior research to satisfactorily address selection has fostered numerous criticism that selection, when unaccounted for, biases influence estimates [23,41]. When interventions are designed around faulty inferences, the social processes they seek to modify are likely to be ineffective. However, our findings suggest that influence and selection are largely independent of one another. Though more research is needed to determine if this finding is generalizable across schools [25], an important implication is that peer influence is a viable intervention lever in some schools even when drinking is simultaneously a basis of friendships.

Most studies assume that selection operates the same for new or existing friendships [26–28]. One contribution of our study is the finding that alcohol selection does not have the same relationship with new versus existing friendships. We found that drinking is less consistently related to continuing existing friendships and is instead more strongly related to forming new friendships. Drinking behaviors in friendship selection do not operate the way most research conceptualizes them and may in fact largely reflect the opportunities that arise through partying rather than a strong preference for drinkers to befriend one another [56]. To the extent that partying reflects novelty and sensation-seeking [57], friendships based on partying would exhibit the pattern we have found: new friendships, but not their continuation. Efforts to channel adolescents into exciting but safer environments may support the creation of new and supportive friendships that protect teens from substance use [58].

In so far as selection is less interpersonal and more environmental, the more amenable it will be to intervention – which is an important finding because prior studies assume that selection is not amenable to intervention. Future work clarifying whether selection operates at the dyad-level or is based on drinking as a “social focus” that organizes social opportunities [59], is thus warranted. Emphasis on friend influence as a policy lever and concern that friend selection is dyadic and not amenable to intervention may have created a false sense that peer selection does not represent a promising avenue for intervention. Our findings suggest that future inventions should continue pursuing strategies that mitigate negative peer influences, while also developing socializing opportunities fostering opportunities to select on healthy behaviors.

Our results also have implications for peer counseling, peer education, and peer-led interventions [60], which have been developed to mixed success [61–64]. Peer-guided approaches typically seek to leverage social network information, such as popularity, to incorporate positive peer influence processes into their design [65]. We found that drinking does not strongly increase popularity, and may damage it as in the large, heavily minority school. Moreover, we found no evidence that drinking is responsive to popularity.

Understanding the local social dynamics of drinking is important as some network processes, such as popularity, differ across schools and population subgroups [66]. The between-school differences likely reflect different attitudes about drinking in majority and minority settings [67,68]. In general, white teens drink more than minority youth [69,70], and the challenges of acquiring alcohol relative to other substances in different settings may decrease its ‘social value’ [71] and therefore the implications it has for socially connecting youth to one another and in fostering popularity. Variation in the role of drinking in promoting popularity and incorporating peer leaders into programs may have disparate implications in different schools where the social status rewards of drinking differ.

Despite limitations (e.g., only two schools), this study makes important contributions to understanding the social context of teen alcohol use. Future work assessing programmatic efforts to prevent teen substance use should incorporate longitudinal network assessments. Friend selection and influence processes are relatively independent when network and behavior change are considered together. Determining how alcohol reduction programs can help teens socialize in venues that foster relationships supportive of positive health behaviors, while also using social networks to encourage positive rather than negative behaviors like drinking, remains to be done. Elucidating these joint processes is critical for ascertaining how programs can be better leveraged to further improve prevention of teen drinking.

## Figures and Tables

**Table 1:** Description of effects included in the models.

Parameter	s_*ik*_ (x,v) X=*network* V=*varname*	Description
*Selection: Covariate parameters*		
Ego (focal adolescent)	V_*i*_ ∑*_j_ x_ij_*	Main effect of adolescent’s *varname* on friend selection (sociability)
Alter (potential friend)	∑*_j_ x_ij_* V_*j*_	Main effect of potential friends’ *varname* on friend selection (popularity)
Ego X alter interaction	V*_i_* ∑*_j_ x_ij_*V_*j*_	Expresses the tendency for adolescents with higher/lower values on *varname* to prefer ties with friends who likewise have higher/lower values relative to the mean (a form of similarity)
Same *varname* (adolescent and potential friend)^1^	∑*_j_ x_ij_ I_j_* (v_*i*_ = v_*j*_)	Effect of the adolescent and the potential friend having an identical value on *varname*
*Selection: Structural parameters*		
Outdegree	∑*_j_ x_ij_*	General tendency to choose a friend
Indegree popularity (sqrt)	$\sum_{j}x_{\text{ij}}\sqrt{x+j}$	Tendency for adolescents with high in-degrees to attract more friends because of their popularity, but where differences between high in-degrees are relatively less important than the same differences between low in-degrees
Reciprocity	∑*_j_ x_ij_ x_ji_*	Tendency to reciprocate a friendship
Transitive triplets	∑*_jh_ x_ih_ x_ij_ x_jh_*	Tendency to be the friend of a friends’ friend
3-Cycles^2^	∑*_jh_ x_ij_ x_jh_ x_hi_*	Tendency for a friend’s friend to choose the adolescent as a friend
*Influence parameters*		
Linear shape effect (*z_i_* = v_*i*_)	*z_i_*	Expresses the basic drive towards high alcohol use values
Quadratic shape effect (*z_i_* = v*_i_*)	$z_{i}^{2}$	Expresses non-linearity in the drive towards higher drinking values
Indegree (*z_i_* = v*_i_*)	*Z_i_* ∑*_j_ x_ji_*	Expresses the tendency for adolescents with high indegrees (who are more popular) to drink more
Outdegree (*z_i_* = v*_i_*)	*Z_i_* ∑ *x_ij_*	Expresses the tendency for adolescents with higher out degrees (who are more ‘active’) to drink more
Average alter (*z_i_* = v*_i_*)	$\frac{z_{i}(\sum_{j}x_{\text{ij}}z_{j})}{\sum_{j}x_{\text{ij}}}$	Positive values indicate that teens whose friends drink more on average themselves also drink more
Covariate effect (*z_i_* ≠ v_*i*_)	*z_i_*v_*i*_	The effect of a covariate (*varname*) on drinking

1 *I* (v*_i_* = v_*j*_) is a function indicating whether v*_i_* = v_*j*_ (=1) or v*_i_* ≠ v*_j_* (= *0*).

2 A positive effect implies generalized reciprocity while a negative effect with a positive transitive triplet effect suggests local hierarchies [53]. Notably, there is a tendency to have a hierarchical ordering with relatively few three-cycles in most friendship networks so that a negative estimate for the three-cycle parameter is usually found [52].

Note: *x* is the network, *i* is the ego or focal adolescent (rows), and *j* is the alter (columns). *v* is a genereic covariate, and *z* is an endogenous behavioral variable (alcohol use, drunkenness frequency).

**Table 2:** Descriptive statistics for variables.

	Total Sample (N=2296)			Sunshine (N=765)		Jefferson (N=1531)	
Variable	N	Mean	(sd)	N	Mean	N	Mean
*Dependent Behavioral Variables*							
Alcohol use, wave 1	1766	2.25	(1.38)	603	2.62	1163	2.05
Alcohol use, wave 2	2293	2.22	(1.39)	765	2.53	1528	2.06
Alcohol use, wave 3	1791	2.23	(1.48)	630	2.62	1161	2.02
Drunkenness, wave 1	1752	1.79	(1.27)	602	2.13	1150	1.61
Drunkenness, wave 2	2292	1.76	(1.24)	764	2.04	1528	1.62
Drunkenness, wave 3	2002	1.82	(1.29)	676	2.17	1326	1.65
*Covariate*							
Off list nominations, wave 1	2296	1.35	(2.32)	765	0.79	1531	1.62
Off list nominations, wave 2	2296	2.19	(2.03)	765	1.69	1531	2.44
Restricted nomination sample, wave 2	2296	0.05	(0.22)	765	0.05	1531	0.05
Female	2296	0.49	(0.50)	765	0.47	1531	0.49
Grade	2273	10.68	(0.94)	756	10.27	1517	10.88
Age	1828	15.96	(1.08)	612	15.74	1106	2.43
Parent education	1791	2.52	(1.12)	685	2.67	1106	2.43
Non-white				765	0.07		
Hispanic/Latino						1529	0.39
African American						1529	0.23
Asian						1529	0.32
White/other						1529	0.06
Regular smoker, wave 1	1764	0.12	(0.32)	605	0.23	1159	0.06
Regular smoker, wave 2	2294	0.22	(0.41)	764	0.35	1530	0.15
Parent alcohol use	1787	1.84	(1.08)	684	2.19	1103	1.63
Alcohol is easy to get	2282	0.30	(0.46)	762	0.32	1520	0.29

**Table 3:** Descriptive network statistics.

	Sunshine			Jefferson		
Wave=	1	2	3	1	2	3
*Baseline*						
Density	0.008	0.005	0.005	0.002	0.001	0.001
Average degree	5.82	4.11	3.47	3.59	2.13	1.75
Number of ties	3399	3059	2162	3551	3100	1927
Missing fraction	0.24	0.03	0.18	0.35	0.05	0.28
Moran’s I^1^	0.28	0.22	0.15	0.19	0.23	0.11
Moran’s I^1^@ distance=2	0.22	0.19	0.19	0.19	0.21	0.20
Number of off list nominations	0.79	1.69	1.60	1.62	2.44	1.84
*Dyad counts*						
Mutual	635	603	433	593	505	287
Asymmetric	1498	1778	1084	1451	1991	1067
Null	168103	274015	192859	486522	1058200	606399
*Jaccard distance*^2^						
Wave 1 ==> 2		0.27			0.21	
Wave 2 ==> 3			0.26			0.22
*Tie changes between observations*	0 => 1	1 => 0	1 => 1	0 => 1	1 => 0	1 => 1
Wave 1 ==> 2	1196	2075	1234	1181	2443	982
Wave 2 ==> 3	1149	1621	949	1083	1588	756
*Alcohol use changes*	Increase	Decrease	No change	Increase	Decrease	No change
Wave 1 ==> 2	174	124	305	249	242	669
Wave 2 ==> 3	157	172	301	251	245	662
*Drunkenness freq. changes*						
Wave 1 ==> 2	112	142	347	153	184	812
Wave 2 ==> 3	210	163	304	235	213	882

1 Moran’s I is a measure of network-attribute autocorrelation (−1 to 1; Moran 1950).

2 The fraction of stable nominations among new, lost, and stable ones during the period [52].

**Table 4:** SAB results for alcohol use frequency in logits.

	Both			Sunshine			Jefferson			
Model/Parameter	b		se	b		se	b		se	t-diff
Models 1 & 2: Independent effects										
Selection: alter	0.002		(0.011)	0.035	*	(0.015)	−0.057	**	(0.021)	**3.565**
Selection: ego	−0.009		(0.012)	−0.013		(0.015)	−0.050		(0.025)	1.269
Selection: ego × alter	0.104	***	(0.008)	0.103	***	(0.014)	0.112	***	(0.016)	−0.423
Influence: average alter	0.136	**	(0.043)	0.266	***	(0.068)	0.182	***	(0.054)	0.967
Model 3: Selection only										
Selection: alter	0.000		(0.011)	0.034	**	(0.013)	−0.065	**	(0.023)	**3.747**
Selection: ego	−0.009		(0.012)	−0.014		(0.015)	−0.047		(0.025)	1.132
Selection: new ego × alter	0.118	***	(0.018)	0.124	***	(0.032)	0.173	***	(0.049)	−0.837
Selection: old ego × alter	0.084	**	(0.026)	0.078	*	(0.033)	0.031		(0.061)	0.678
Model 4: Selection+influence										
Selection: alter	−0.002		(0.011)	0.034	*	(0.015)	−0.065	**	(0.024)	**3.498**
Selection: ego	−0.010		(0.012)	−0.017		(0.020)	−0.050		(0.025)	1.031
Selection: new ego × alter	0.118	***	(0.017)	0.127	***	(0.028)	0.174	***	(0.035)	−1.049
Selection: old ego × alter	0.086	***	(0.023)	0.079	*	(0.035)	0.033		(0.039)	0.878
Influence: average alter	0.137	**	(0.049)	0.265	***	(0.076)	0.184	**	(0.063)	0.821
Model 5:+network controls										
Selection: alter	0.004		(0.010)	0.024		(0.014)	−0.037		(0.023)	**2.265**
Selection: ego	−0.020		(0.013)	−0.015		(0.015)	−0.056	*	(0.028)	1.291
Selection: new ego × alter	0.107	***	(0.016)	0.113	***	(0.028)	0.157	***	(0.024)	−1.193
Selection: old ego × alter	0.075	**	(0.024)	0.057	*	(0.028)	0.053		(0.041)	0.081
Influence: in degree	−0.012		(0.016)	0.003		(0.019)	−0.031		(0.027)	1.030
Influence: out degree	0.077	***	(0.020)	0.008		(0.025)	0.054		(0.034)	−1.090
Influence: average alter	0.166	***	(0.050)	0.278	***	(0.070)	0.215	**	(0.076)	0.610
Model 6:+covariates										
Selection: alter	0.007		(0.010)	0.033	*	(0.016)	−0.038		(0.025)	**2.392**
Selection: ego	−0.015		(0.016)	0.001		(0.017)	−0.049		(0.033)	1.347
Selection: new ego × alter	0.098	***	(0.019)	0.077	*	(0.033)	0.132	***	(0.039)	−1.077
Selection: old ego × alter	0.039		(0.025)	0.074	*	(0.035)	0.031		(0.056)	0.651
Influence: in degree	−0.005		(0.015)	0.007		(0.017)	−0.031		(0.028)	1.160
Influence: out degree	0.032		(0.019)	0.013		(0.024)	0.044		(0.037)	−0.703
Influence: average alter	0.176	***	(0.046)	0.221	**	(0.072)	0.170	*	(0.072)	0.501

Standard errors in second column

* p<0.05,

** p<0.01,

*** p<0.001

**Table 5:** SAB results for drunkenness frequency in logits.

	Both			Sunshine			Jefferson			
Model/Parameter	b		se	b		se	b		se	t-diff
Models 1 and 2: Independent effects										
Selection: alter	−0.040	*	(0.016)	−0.004		(0.016)	−0.113	**	(0.038)	**2.644**
Selection: ego	−0.026		(0.023)	−0.028		(0.019)	−0.060		(0.048)	0.620
Selection: ego × alter	0.126	***	(0.013)	0.109	***	(0.014)	0.131	***	(0.028)	−0.703
Influence: average alter	0.117	*	(0.047)	0.368	***	(0.067)	0.215	***	(0.049)	1.843
Model 3: Selection only										
Selection: alter	−0.039	*	(0.018)	−0.009		(0.018)	−0.124	**	(0.040)	**2.622**
Selection: ego	−0.025		(0.018)	−0.030		(0.020)	−0.059		(0.034)	0.735
Selection: new ego × alter	0.114	***	(0.018)	0.154	***	(0.041)	0.185	***	(0.042)	−0.528
Selection: old ego × alter	0.144	***	(0.033)	0.054		(0.045)	0.056		(0.053)	−0.029
Model 4: Selection+influence										
Selection: alter	−0.042		(0.028)	−0.012		(0.044)	−0.128		(0.083)	1.235
Selection: ego	−0.028		(0.021)	−0.035		(0.027)	−0.063		(0.035)	0.633
Selection: new ego × alter	0.115	***	(0.028)	0.159	**	(0.055)	0.189	***	(0.041)	−0.437
Selection: old ego × alter	0.142		(0.085)	0.055		(0.052)	0.053		(0.072)	0.023
Influence: average alter	0.118		(0.066)	0.366	*	(0.154)	0.218	***	(0.060)	0.895
Model 5:+network controls										
Selection: alter	−0.018		(0.018)	−0.007		(0.018)	−0.067		(0.166)	0.359
Selection: ego	−0.035	*	(0.015)	−0.030		(0.022)	−0.066		(0.059)	0.572
Selection: new ego × alter	0.102	**	(0.032)	0.147	***	(0.034)	0.152		(0.319)	−0.016
Selection: old ego × alter	0.099	***	(0.028)	0.018		(0.043)	0.086		(0.067)	−0.854
Influence: in degree	0.016		(0.044)	0.031		(0.016)	−0.014		(0.036)	1.142
Influence: out degree	0.061		(0.055)	−0.026		(0.021)	0.028		(0.040)	−1.195
Influence: average alter	0.153		(0.094)	0.363	***	(0.076)	0.236		(0.367)	0.339
Model 6: +covariates										
Selection: alter	−0.010		(0.017)	0.001		(0.020)	−0.054		(0.049)	1.039
Selection: ego	−0.015		(0.015)	−0.013		(0.026)	−0.044		(0.101)	0.297
Selection: new ego × alter	0.107	**	(0.032)	0.111	***	(0.030)	0.121		(0.065)	−0.140
Selection: old ego × alter	0.020		(0.038)	0.031		(0.039)	0.056		(0.071)	−0.309
Influence: in degree	0.016		(0.019)	0.027		(0.021)	−0.011		(0.041)	0.825
Influence: out degree	0.014		(0.026)	0.001		(0.026)	0.016		(0.041)	−0.309
Influence: average alter	0.219	***	(0.049)	0.281	***	(0.085)	0.211		(0.141)	0.425

Standard errors in second column

* p<0.05,

** p<0.01,

*** p<0.001
